# Supplementary material for: Observation of Bloch oscillations in complex PT-symmetric photonic lattices
Source: Sci Rep. 2015 Dec 7;5:17760. doi: 10.1038/srep17760 (PMC4671013; doi:10.1038/srep17760)
Supplement: Supplementary Materials [file srep17760-s1.pdf]

Supplementary Information

Observation of Bloch oscillations in complex PT-symmetric photonic lattices

Martin Wimmer[1,2], Mohammed-Ali Miri[3], Demetrios Christodoulides[3], *Ulf Peschel[4]

[1]Institute of Optics, Information and Photonics, Friedrich-Alexander-Universität Erlangen-Nürnberg, Staudtstraße 7/B2, 91058 Erlangen, Germany

[2]Erlangen Graduate School in Advanced Optical Technologies (SAOT), 91058 Erlangen, Germany

[3]CREOL, College of Optics and Photonics, University of Central Florida, Orlando, Florida 32816–2700, USA

[4]Institute of Solid State Theory and Optics, Friedrich Schiller University Jena, Max-Wien-Platz 1, 07743 Jena

*Correspondence and requests for materials should be addressed to U.P. (email: ulf.peschel@uni-jena.de)

Supplementary Figures:

Supplementary Figure 1:

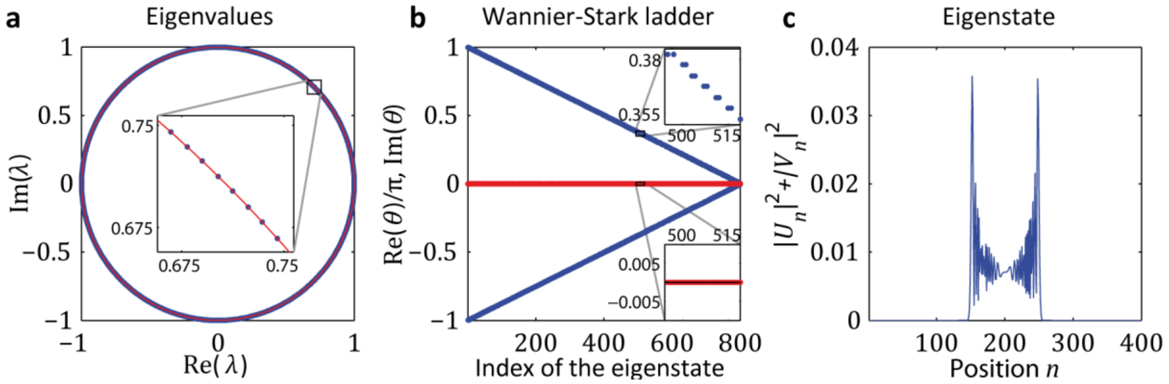

Supplementary Figure 1 | Localized eigenstates of the Hermitian lattice with a transversal Bloch gradient. a, For a transversal Bloch gradient $\varphi_0 = \frac{\pi}{200}$ and a Hermitian lattice of size $N = 400$, all eigenvalues λ are located on the unit circle in the complex plane. The corresponding energy levels form a Wannier-Stark ladder (**b**) with the corresponding eigenstates shown in (**c**). Periodic boundaries are assumed. Please see also Supplementary Note 2.

Supplementary Figure 2:

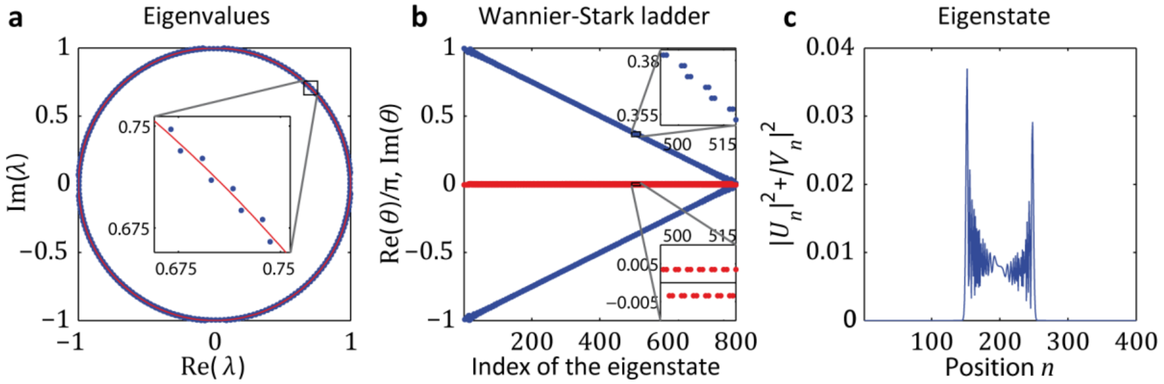

Supplementary Figure 2 | Localized eigenstates of the global PT-symmetric lattice with a transversal Bloch gradient. Again as in Supplementary Fig. 1**,** corresponding eigenvalues λ and eigenstates are shown in (a) and (c). The Wannier-Stark ladder is depicted in (b). In contrast to the Hermitian System, the imaginary part of the propagation constant θ is small but not vanishing for an arbitrary Bloch gradient φ_0. Thus, the eigenvalues are shifted with respect to the unit circle of the complex plain. Like in Supplementary Fig. 2 the Bloch gradient was chosen to be $\varphi_0 = \frac{\pi}{200}$, together with a gain factor of $G = 1.1$ and a lattice size $N = 400$ with periodic boundaries.

Supplementary Figure 3:

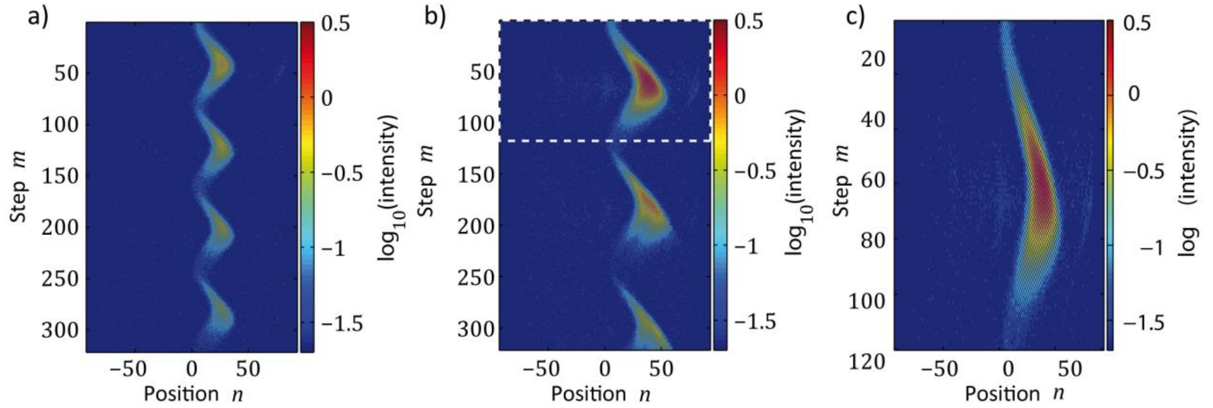

Supplementary Figure 3 | Local PT-symmetric Bloch oscillations over several periods. Experimental Bloch oscillations for gradients $\varphi_0 = \frac{\pi}{20}$ (**a**) and $\varphi_0 = \frac{\pi}{30}$ (**b**) are shown for a local PT-symmetric configuration with an amplifying factor $G = 1.1$. The dashed white rectangle in (**b**) shows the data used in (**c**) and in Figure 5 of the main text.

Supplementary Notes

Supplementary Note 1: Spatio-temporal transformation of Bloch gradients.

For discrete time quantum walks it is possible to replace a transversal (spatial) Bloch gradient with a temporal phase modulation [1,2]. By starting with a purely spatial phase modulation

$$\tilde{u}_n^m = \frac{1}{\sqrt{2}}(\tilde{u}_{n+1}^m + i\tilde{v}_{n+1}^m)e^{\frac{in\varphi}{2}} \qquad (1.1)$$

$$\tilde{v}_n^m = \frac{1}{\sqrt{2}}(\tilde{v}_{n-1}^m + i\tilde{u}_{n-1}^m)e^{\frac{in\varphi}{2}} \qquad (1.2)$$

and applying a transformation of the form

$$\tilde{u}_n^m = u_n^m e^{+\frac{inm\varphi}{2}} e^{-\frac{im^2\varphi}{4}} e^{+\frac{im\varphi}{4}} \qquad (2.1)$$

$$\tilde{v}_n^m = v_n^m e^{+\frac{inm\varphi}{2}} e^{-\frac{im^2\varphi}{4}} e^{+\frac{im\varphi}{4}} \qquad (2.2)$$

one reaches at the final modulation scheme along the temporal axis, as it is used in the manuscript

$$u_n^{m+1} = \frac{1}{\sqrt{2}}(u_{n+1}^m + iv_{n+1}^m)e^{+im\varphi} \qquad (3.1)$$

$$v_n^{m+1} = \frac{1}{\sqrt{2}}(v_{n-1}^m + iu_{n-1}^m). \qquad (3.2)$$

Supplementary Note 2: Wannier-Stark ladder for Bloch oscillations in the global PT-symmetric system

For a temporal modulation, any initial state is subject to a continuous shift through the Brillouin zone as pointed out in the main part. However, one can still find stationary eigenstates of the system, by choosing the spatio-temporal transformation mentioned in Supplementary Note 1. This conjunction allows one, to move from a temporal driven system of nonstationary states to a spatial modulation scheme, which possess again stationary eigenstates as it is shown in the following:

In case of global PT symmetry, the evolution equations

$$u_n^{m+1} = \frac{\sqrt{G_\mathrm{u}}}{\sqrt{2}} \left(u_{n+1}^m + i v_{n+1}^m\right) e^{i\varphi n} \tag{4.1}$$
$$v_n^{m+1} = \frac{\sqrt{G_v}}{\sqrt{2}} \left(v_{n-1}^m + i u_{n-1}^m\right) e^{i\varphi n} \tag{4.2}$$

for a single time step with a spatial phase modulation correspond to the set of equations

$$2u_n^{m+2} = \left(u_{n+2}^m + i v_{n+2}^m + iG^{-1} v_n^m - G^{-1} u_n^m\right) e^{i\varphi(2n+1)} \tag{5.1}$$
$$2v_n^{m+2} = \left(v_{n-2}^m + i u_{n-2}^m + iG u_n^m - G v_n^m\right) e^{i\varphi(2n-1)} \tag{5.2}$$

for a double step. Note, that in this case gain and loss are balanced ($G = G_\mathrm{u} = G_\mathrm{v}^{-1}$), and are furthermore exchanged after every roundtrip.

Numerical calculations (see Supplementary Fig. 1) predict the existence of a Wannier-Stark ladder in both the Hermitian case ($G = 1$) and the broken PT-symmetric case ($G \neq 1$, see Supplementary Fig. 2).

In order to search numerically for eigenstates, a stationary ansatz of the form

$$\begin{pmatrix} u_n^m \\ v_n^m \end{pmatrix} = \begin{pmatrix} U_n \\ V_n \end{pmatrix} e^{i\theta m} \tag{6}$$

is inserted into Supplementary Eqs. 5.1 and 5.2. The propagation constant θ is directly linked to the eigenvalues $\lambda = e^{i\theta}$ of the eigenstates $(U_n, V_n)^t$.

Supplementary Note 3: Derivation of the band structure of the local PT-symmetric system.

The band structure of the local PT-symmetric system in Eq. 5 is derived by starting with the evolution equations

$$u_n^{m+1} = \frac{1}{\sqrt{2G}}(u_{n+1}^m + \mathrm{i}v_{n+1}^m), \qquad (7.1)$$

$$v_n^{m+1} = \frac{\sqrt{G}}{\sqrt{2}}(v_{n-1}^m + \mathrm{i}u_{n-1}^m), \qquad (7.2)$$

and inserting the plane wave ansatz from Eq. 2 in the main text into Eqs. 1.1 and 1.2. The resulting linear equation system

$$\begin{pmatrix} -\sqrt{2}e^{i\theta} + \frac{1}{\sqrt{G}}e^{iQ} & \frac{ie^{iQ}}{\sqrt{G}} \\ i\sqrt{G}e^{-iQ} & -\sqrt{2}e^{i\theta} + \sqrt{G}e^{-iQ} \end{pmatrix}\begin{pmatrix} U \\ V \end{pmatrix} = 0 \qquad (8)$$

can be solved for the dispersion relation between θ and Q by demanding that the determinant

$$\det\begin{vmatrix} -\sqrt{2}e^{i\theta} + \frac{1}{\sqrt{G}}e^{iQ} & \frac{ie^{iQ}}{\sqrt{G}} \\ i\sqrt{G}e^{-iQ} & -\sqrt{2}e^{i\theta} + \sqrt{G}e^{-iQ} \end{vmatrix} = 0. \qquad (9)$$

is vanishing. For convenience, the gain factor G is replaced by $\gamma = \log G$. The resulting expression

$$\left(-\sqrt{2}e^{i\theta} + e^{i\left(Q+\frac{i}{2}\gamma\right)}\right)\left(-\sqrt{2}e^{i\theta} + e^{-i\left(Q+\frac{i}{2}\gamma\right)}\right) + 1 = 0 \qquad (10)$$

is first simplified

$$2e^{i\theta} - \sqrt{2}\left(e^{i\left(Q+\frac{i}{2}\gamma\right)} + e^{-i\left(Q+\frac{i}{2}\gamma\right)}\right) + 2e^{-i\theta} = 0 \qquad (11)$$

And then solved for $\cos\theta = \frac{1}{\sqrt{2}}\cos\left(Q + \frac{i}{2}\gamma\right)$.

Supplementary References:

1. Matjeschk, R. et al. Quantum Walks with Nonorthogonal Position States. *Phys. Rev. Lett.* **109,** 240503 (2012).
2. Regensburger, A. et al. Photon Propagation in a Discrete Fiber Network: An Interplay of Coherence and Losses. *Phys. Rev. Lett*. **107**, 233902 (2011).
